# Diagnostic test accuracy of a novel smartphone application for the assessment of attention deficits in delirium in older hospitalised patients: a prospective cohort study protocol

**DOI:** 10.1186/s12877-018-0901-5

**Published:** 2018-09-17

**Authors:** Lisa-Marie Rutter, Eva Nouzova, David J. Stott, Christopher J. Weir, Valentina Assi, Jennifer H. Barnett, Caoimhe Clarke, Nikki Duncan, Jonathan Evans, Samantha Green, Kirsty Hendry, Meigan McGinlay, Jenny McKeever, Duncan G. Middleton, Stuart Parks, Robert Shaw, Elaine Tang, Tim Walsh, Alexander J. Weir, Elizabeth Wilson, Tara Quasim, Alasdair M.J. MacLullich, Zoë Tieges

**Affiliations:** 1 Edinburgh Delirium Research Group, University of Edinburgh, Edinburgh, UK; 2 Institute of Cardiovascular and Medical Sciences, University of Glasgow, Glasgow, UK; 3 Edinburgh Clinical Trials Unit, Usher Institute of Population Health Sciences and Informatics, University of Edinburgh, Edinburgh, UK; 4 Cambridge Cognition Ltd, Cambridge, UK; 5 Department of Psychiatry, University of Cambridge, Cambridge, UK; 6 Institute of Health and Wellbeing, University of Glasgow, Glasgow, UK; 7 Medical Devices Unit, West Glasgow Ambulatory Care Hospital, Glasgow, UK; 8 Critical Care Medicine and Anaesthesia, University of Edinburgh, Edinburgh, UK; 9 Critical Care Medicine and Anaesthesia, Royal Infirmary of Edinburgh, Edinburgh, UK; 10 Anaesthesia, Critical Care and Pain Medicine, Glasgow Royal Infirmary, Glasgow, UK; 11 Centre for Cognitive Ageing and Cognitive Epidemiology, University of Edinburgh, Edinburgh, UK

**Keywords:** Delirium, Dementia, Attention, Neuropsychological test, Cognition, Consecutive series, Smartphone test, Diagnostic accuracy study, Prospective study

## Abstract

**Background:**

Delirium is a common and serious clinical syndrome which is often missed in routine clinical care. The core cognitive feature is inattention. We developed a novel bedside neuropsychological test for assessing inattention in delirium implemented on a smartphone platform (DelApp). We aim to evaluate the diagnostic performance of the DelApp in a representative cohort of older hospitalised patients.

**Methods:**

This is a prospective study of older non-scheduled hospitalised patients (target *n* = 500, age ≥ 65), recruited from elderly care and acute orthopaedic wards. Exclusion criteria are: non-English speakers; severe vision or hearing impairment; photosensitive epilepsy.

A structured reference standard delirium assessment based on DSM-5 criteria will be used, which includes a cognitive test battery administered by a trained assessor (Orientation-Memory-Concentration Test, Abbreviated Mental Test-10, Delirium Rating Severity Scale-Revised-98, digit span, months and days backwards, Vigilance A’ test) and assessment of arousal (Observational Scale of Level of Arousal, Richmond Agitation Sedation Scale). Prior change in cognition will be documented using the Informant Questionnaire on Cognitive Decline in the Elderly. Patients will be categorized as delirium (with/without dementia), possible delirium, dementia, no cognitive impairment, or undetermined.

A separate assessor (blinded to diagnosis and assessments) will administer the DelApp index test within 3 h of the reference standard assessment. The DelApp comprises assessment of arousal (score 0-4) and sustained attention (score 0-6), yielding a total score between 0 and 10 (higher score = better performance). Outcomes (length of stay, mortality and discharge location) will be collected at 12 weeks.

We will evaluate a priori cutpoints derived from a previous case-control study. Measures of the accuracy of DelApp will include sensitivity, specificity, positive and negative predictive values, and area under the ROC curve. We plan repeat assessments on up to 4 occasions in a purposive subsample of 30 patients (15 delirium, 15 no delirium) to examine changes over time.

**Discussion:**

This study evaluates the diagnostic test accuracy of a novel smartphone test for delirium in a representative cohort of older hospitalised patients, including those with dementia. DelApp has the potential to be a convenient, objective method of improving delirium assessment for older people in acute care.

**Trial registration:**

Clinical trials.gov, NCT02590796. Registered on 29 Oct 2015. Protocol version 5, dated 25 July 2016.

**Electronic supplementary material:**

The online version of this article (10.1186/s12877-018-0901-5) contains supplementary material, which is available to authorized users.

## Background

### Delirium in hospitalised older patients

Delirium is a common and severe neuropsychiatric syndrome characterized by acute and fluctuating impairments in arousal, attention and cognition. It affects at least 1 in 8 general hospital patients [1–3] and is commonly triggered by acute illness, trauma or medications. It is independently associated with poor outcomes including higher morbidity, mortality, longer hospital stay, and an increased risk of new or accelerated cognitive impairment [4–6]. Moreover, delirium is often distressing for patients and their carers [7]. The economic impact of delirium is also large [8].

Despite this, delirium remains poorly detected in hospital with at least two-thirds of cases missed [1]. This failure to detect delirium remains a major barrier to improving care, because accurate diagnosis is crucial to ensure prompt treatment of causes, management of risk (e.g. falls, dehydration) and relief of distress [9]. Therefore, as stated in major national guidelines and policy documents, improving the detection of delirium is a priority for the NHS and other healthcare systems [10, 11].

### Neuropsychology of inattention in delirium

The core diagnostic feature of delirium is inattention [12]. There is significant overlap between the phenomenology of delirium and other major differential diagnoses including dementia, but inattention is important diagnostically because it is usually much less prominent in dementia [13–15]. Differentiating between delirium and dementia is key to optimal treatment and management of the symptoms and underlying causes of these syndromes.

Although the extent to which different aspects of attention are affected in delirium is poorly understood, there is some evidence to suggest that the ability to maintain attention to stimuli over time (i.e. sustained attention) is consistently impaired [13]. This could help explain the range of performance deficits seen in delirium across a variety of neuropsychological tests, since staying alert and sustaining attention to the stimulus material is a requirement of most tests.

Inattention in delirium is currently detected using either subjective interview-based assessments, a range of cognitive tests (e.g. digit span, months of the year backwards), or a combination of both. Subjective assessments and judgements of attention often have inadequate reliability and require specialist or trained assessors [16]. Most existing tests of attention have not been formally validated for delirium detection in representative samples of patients with a broad spectrum of cognitive and behavioural impairments [17] and are usually not specific to delirium (i.e. they discriminate poorly between delirium and other mental disorders including dementia [18]). This is likely in part because many so-called ‘attention’ tests measure a range of other cognitive functions including memory and executive function which are known to be also impaired in dementia [19].

In summary, currently available assessments of inattention have several drawbacks including inadequate discrimination between delirium and dementia and subjectivity. The lack of well-validated objective tools for attention assessment in delirium leads to uncertainty over diagnosis, and is likely a major contributing factor in the low detection rates of delirium.

### Rationale for the study

To help address the need for objective bedside tests of attention suitable for use in older hospitalised patients with delirium and/or dementia, we previously developed a neuropsychological test of sustained and focused attention implemented on a purpose-built computerised device called the Edinburgh Delirium Test Box (EDTB; [13, 20, 21]). We subsequently developed a prototype software application for smartphones (‘DelApp’) which is based on the EDTB tasks [22]. Some severely impaired patients cannot engage with verbal cognitive testing, and to take account of this the DelApp incorporates an initial brief assessment of level of arousal, involving observations and participant’s responses to a sequence of simple commands.

A pilot single-rater case-control study using the DelApp showed significantly lower scores in patients with delirium compared to groups with dementia or no cognitive impairment. Receiver Operating Characteristic (ROC) curve analyses found excellent accuracy of the DelApp for discriminating between delirium and dementia and between delirium and cognitively normal controls [22]. These pilot study findings are promising, suggesting that the DelApp test merits further assessment as a method for objective assessment of attention in delirium.

### Study aims and objectives

The *primary objective* is to evaluate the diagnostic accuracy of the DelApp in a representative sample of patients versus the reference standard. A priori cut points for discriminating delirium from the whole sample and from dementia, derived from preceding case-control studies [23], will be evaluated.

The *secondary objectives* are:

A. To evaluate the DelApp total score as a measure of delirium severity (construct validity);
B. To determine the association between DelApp total scores and other measures of attention (i.e. months of the year backward, days of the week backward, Vigilance A’ test and counting down from 20 to 1) (construct validity);
C. To assess if DelApp scores predict important clinical outcomes: length of stay, mortality at 12 weeks and discharge destination;
D. To determine if DelApp scores are responsive to changes in delirium status and severity for a person over time.
E. To assess inter-rater agreement of the DelApp assessment.

## Methods

### Study overview

**Fig. 1:**
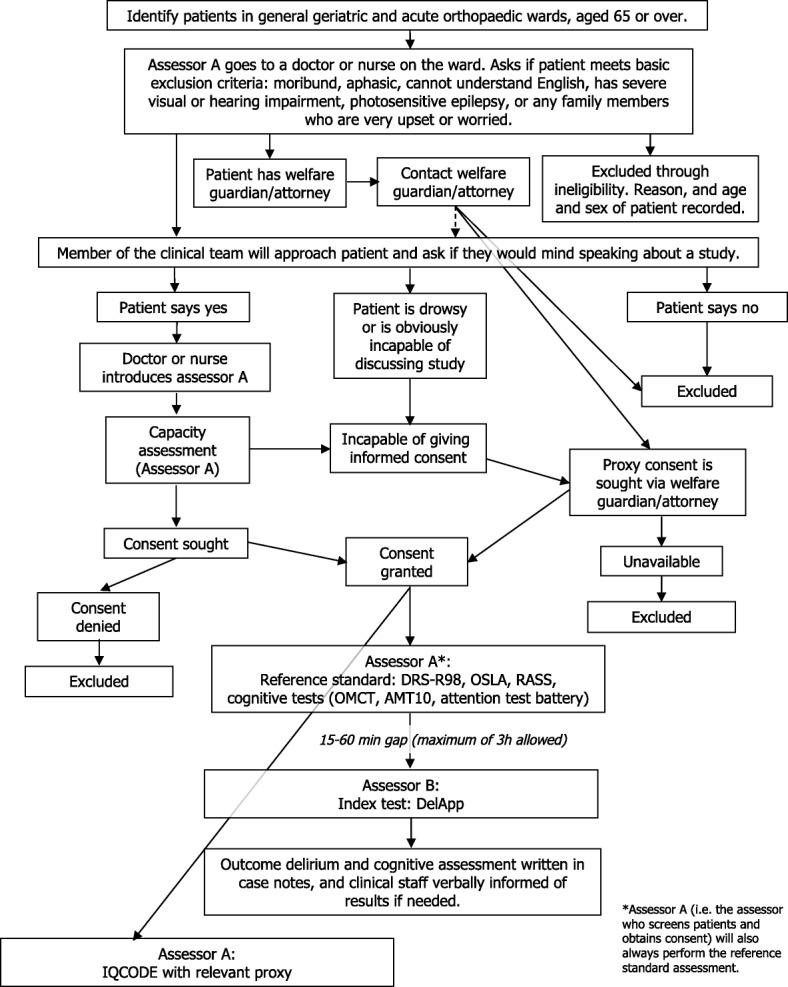
DelApp study design overview flowchart

This is a diagnostic accuracy study involving a representative sample of patients recruited from general and acute medical hospital wards. We aim to recruit 500 patients aged 65 years and over from the Royal Infirmary of Edinburgh and the Glasgow Royal Infirmary, Scotland, UK. We expect that at least 100 patients will have delirium [3], and a similar or greater number will have dementia without delirium [24]. All patients will be screened in order of arrangement of beds on the respective wards with no limit on duration of patient stay, subject to researcher availability. Informed consent will be sought from patients or their relatives or welfare guardian. The study overview flowchart is shown in Fig. 1.

The DelApp (index test) will be evaluated against the reference standard diagnosis of delirium. The index test and reference standard assessment will be administered to the same patients by two independent, trained assessors. The second assessor who administers the index test will be blinded to the reference standard assessment, the patients’ diagnosis and other clinical information to ensure unbiased scoring of the DelApp. Total test time will be around 15-25 min, with a target interval of 15-60 min between index and reference standard assessments and a maximum possible interval of 3 h.

A purposive subsample of 30 patients (i.e. 15 with delirium at any of the assessments, and 15 without delirium) will be assessed on up to three further occasions at least 1 day apart, along with the reference standard, to evaluate changes in DelApp scores and the reference standard assessment over time. Inter-rater assessments of reference standard and index test are planned in 40 patients (20 per recruitment site).

The recruitment period lasted from August 2016 to March 2018. Data cleaning and data checks are ongoing as of April 2018.

### Participants

#### Inclusion criteria

Participants will be included if they are aged 65 years or over and if they have capacity to provide written, informed consent or if a suitable relative or welfare guardian/attorney is available to provide informed consent on their behalf.

#### Exclusion criteria

Participants will be excluded if they have vision or hearing impairment severe enough to preclude testing or interview or a history of photosensitive epilepsy, or if they are unable to understand English.

### Informed consent and enrolment

Potentially eligible patients will be identified through discussion of the inclusion and exclusion criteria with a medical staff member responsible for the care of the patient. The researcher will also gather information from the medical staff member about the patient (using a formal checklist) to check for potential issues including if the patient has deteriorated since first being identified as eligible, whether the patient is participating in other research and any concerns the family or carers have. If they are extremely concerned about the patient’s health or the care that the patient receives, the patient will not be approached.

Eligible patients will first be approached by a member of the patient’s clinical care team, who will inform them that a researcher would like to talk to them about taking part in a study. If the patient expresses an interest in taking part then the clinical team member will introduce the researcher to the patient. Informed consent will be sought by the researcher, who is trained in conducting capacity assessment and obtaining consent. Patients will be given an information sheet and have the opportunity to read it and ask questions. If the researcher judges that the patient lacks capacity to provide informed consent, proxy consent will be sought from the nearest relative or welfare guardian/attorney at the first opportunity in person, or by telephone.

### Index test: DelApp

The DelApp (index test) comprises a brief arousal assessment followed by a sustained attention task whereby participants are instructed to count a number of stars presented serially on a smartphone screen.

The arousal assessment consists of the following items: (1) judge whether the patient is awake and responsive, or if the patient opens their eyes to speech or a touch on the shoulder for more than 10 s (2 points) or less than 10 s (1 point); (2) ask the patient to say their name or obey a one-stage command (e.g. lifting one arm) (1 point); and (3) ask the patient to follow an object with their eyes for 5 s (1 point). This yields a maximum possible score of 4 indicating that the patient is awake and able to follow basic commands. Participants who achieve a score of ≥3 on the arousal assessment proceed with the attention task. In case of an arousal score below 3, the assessment ends and the participant receives a total DelApp score based on the arousal assessment alone. Assessor instructions for this assessment and a schematic display of items and scoring procedures are presented in Additional file 1: Table S1 and Figure S1, respectively.

**Fig. 2:**
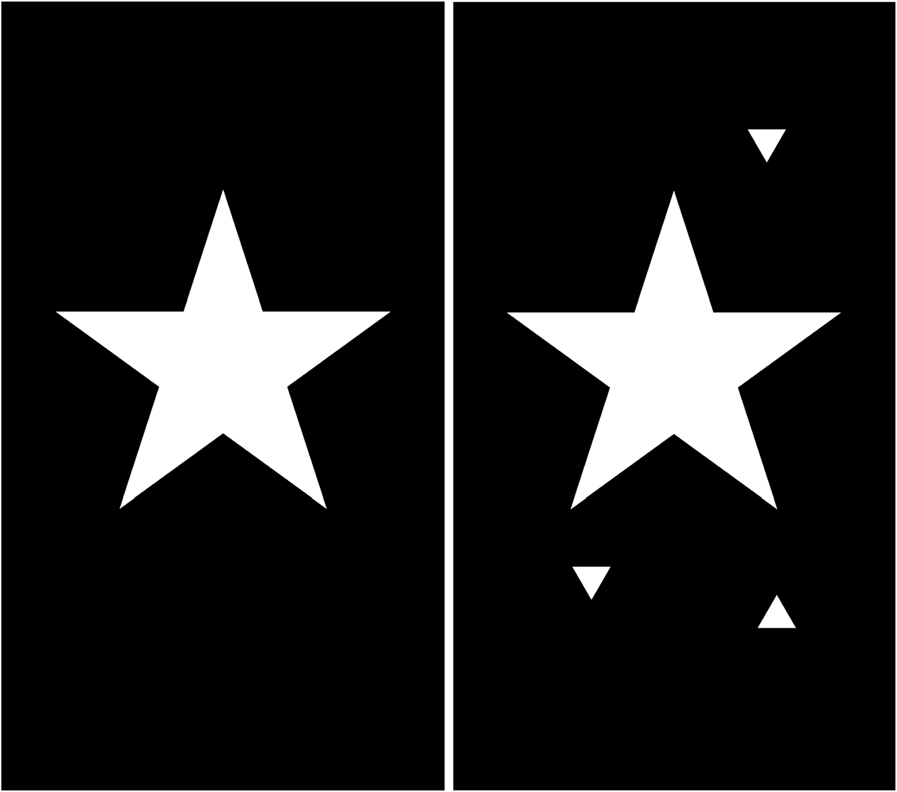
Examples of stimuli used in the DelApp attention test: five-pointed star without distracting shapes (left; trials 1-3) and with surrounding distracter shapes (right; trials 4-7)

The attention task requires counting a series of large white five-pointed stars (approximately 5 cm diameter) appearing on the screen presented against a black background (Fig. 2). As the task progresses, distracting triangular shapes appear around the stimuli and the time delay between stars increases, placing a greater demand on the participant’s attention. The counting task consists of 7 trials, the first being a practice trial which is not scored. Stars and distracting shapes are presented for a fixed duration of 1000 msec each. The number of stars and time delays between the stars vary based on a pre-defined design. The number of stars presented on a given trial ranges between 2 and 5 stars for trials 1-3, between 4 and 6 stars for trials 4 and 5, and between 6 and 8 stars for trials 6 and 7. The time delays between stars vary within the range of 1500-3000 msec for trials 1-5 and 3000-4500 msec for trials 6 and 7. Distracting shapes are presented in trials 4-7 only, with approximately twice as many distracting shapes presented in trials 6-7 compared to trials 4-5.

Prior to the start of the counting task, participants perform a brief visual pre-test whereby they are asked to identify a single star presented on the smartphone screen, to check for visual impairment severe enough to affect performance on the counting task. Testing will proceed only if the patient correctly identifies the shape. Assessor instructions and scoring procedures for the DelApp visual pre-test and attention assessment are presented in Additional file 1: Table S1.

The DelApp assessment is usually completed within 5 min, but the time varies between participants. Trials are scored as correct or incorrect. A missing answer is considered an incorrect response. If the participant gives an incorrect answer (or no answer) twice in a row, the assessment ends. Total DelApp score consists of the arousal score (score 0-4) and attention score (score 0-6), yielding a total score between 0 and 10 whereby a score of 10 indicates normal attention.

### Reference standard assessment

The reference standard assessment consists of cognitive and delirium assessment tools and observational scales, supplemented by inspection of the case notes, discussion with the clinical team and an informant history if available.

To assess overall cognitive function (including memory and attention), the Short Orientation-Memory-Concentration Test (OMCT; [25]) and abbreviated mental test (AMT10 [26]) will be administered. Attentional function will be measured with a brief attentional test battery consisting of digit span (forward and backward), months of the year backward (as part of the OMCT), days of the week backward and the Vigilance ‘A’ test from the Montreal Cognitive Assessment [27]. Subjective feelings of pain at the time of assessment will be assessed with a 10-point pain thermometer [28].

The Delirium Rating Scale-Revised-98 (DRS-R98 [29]) will be used to assess delirium severity, which requires inspection of case notes, discussions with staff and additional cognitive testing of short- and long-term memory and visuospatial function (which are not adequately captured in the OMCT or AMT10). For details about assessor instructions and scoring procedures of the neuropsychological tests used in the reference standard assessment, see Additional file 2.

Level of arousal is assessed with the Observational Scale of Level of Arousal (OSLA [20]) and the Richmond Agitation-Sedation Scale in which the expression “sedated” is replaced by “drowsy” [30]. These observational scales require no extra time with the patient and are scored directly after the cognitive assessments. Any formal prior diagnosis of dementia will be recorded and, if available, a relative or other individual who has known the patient for 10 years will be asked to fill out the Informant Questionnaire on Cognitive Decline in the Elderly (IQCODE; [31]) to help determine whether patients had longstanding cognitive decline prior to their hospital admission. Ascertainment of DSM-5 delirium will be based on a standardised process using operationalised DSM-5 criteria which take into account all available information (i.e. cognitive test scores, observations, case notes etc.; Table 1).

**Table 1:** DSM-5 structured reference standard for delirium

DSM-5 criteria	Source of information			
	Cognitive test	Arousal scale	Interview	Informant
A. Disturbance in attention and awareness	• Months of the year backward • Days of the week backwards • Counting from 20 to 1 • Digit span forward (3, 4 and 5 digits) • Vigilance A Inattention if errors on ≥2 of the tests above.	• RASS (score ≠ 0) • OSLA (score > 2)	Any behavioural signs that suggest inattention, lack of awareness and orientation, distractibility, verbal perseverations, etc.	Evidence of inattention from an informant (clinical staff, medical notes, relatives) within the last hour.
B. Acute change from baseline cognitive status (usually hours to a few days) and/or fluctuation in symptom severity	N/A	N/A	N/A	Evidence of acute onset and/or fluctuation in symptom severity from medical notes, clinical staff or relatives. Has there been a sudden change in mental state, diurnal variation or altered level of consciousness during the day?
C. Additional disturbance in cognition	MEMORY • Recall of 3 words (lemon, key, ball) • Recall of address (AMT10) • Recollection of World War 2 start and end date (AMT10) • Name of current prime minister (AMT10) Memory impairment if error on any of the tests above. ORIENTATION (from OMCT) • Time • Person • Place Disorientation present if error on ≥2 items.	N/A	LANGUAGE Assess fluency, grammar, comprehension and semantic content during communication. PERCEPTUAL DISTURBANCE Self-report of hallucination and/or delusions VISUOSPATIAL Ask patient which of any two objects is closer to them; Ask the patient if the room looks tilted; Consider placement of initials and signature on consent form.	Any evidence of cognitive disturbance that is obtained from medical notes or clinical team.
D. Disturbances in criteria A and C are not better explained by another disorder and do not occur in the context of coma	N/A	N/A	N/A	Discuss with medical team and/or family and consult medical notes.
E. Evidence that the disturbance is a direct physiologic consequence of another medical condition, substance intoxication or withdrawal, exposure to a toxin or because of multiple etiologies.	N/A	N/A	N/A	Discuss with medical team and/or family and consult medical notes.

*DSM-5* Diagnostic Statistical Manual-5, *AMT10* Abbreviated Mental Test 10, *OMCT* Short Orientation, Memory and Concentration Task, *OSLA* Observational Scale of Level of Arousal, *RASS* Richmond Agitation-Sedation Scale

In view of the transient and fluctuating nature of delirium, we expect that for some patients it will be challenging to determine whether or not they meet all DSM-5 criteria for delirium. These challenging cases will be discussed in regular group discussions with a delirium expert (AMJM or DJS) who will be blinded to the index test scores (DelApp).

### Grouping of participants

Patients will be categorised into the following groups: delirium (with or without dementia), possible delirium, dementia without delirium, no cognitive impairment, and undetermined.

Patients will be included in the delirium group if (1) there is evidence of inattention and other cognitive problems from the reference standard which cannot be better attributed to pre-existing cognitive impairment, and/or (2) if there is positive evidence of delirium in the 24 h prior to the reference standard assessment in the case notes or as indicated by a senior member of the clinical team. If patients display *some* symptoms of delirium, but there is uncertainty about diagnosis due to missing information (e.g. it is unclear if the problems represent an acute change from baseline cognitive status, because no informant is available) then these patients will be grouped as having *possible* delirium.

Patients who have a formal diagnosis of dementia and/or an IQCODE score > 3.82 [32], with no evidence of delirium (specifically no acute change from baseline cognitive status) will be grouped as having dementia.

Patients who show no inattention or other cognitive problems as indicated by an OMCT score > 20 *and* an AMT10 score > 7 and have no acute change from baseline are grouped as having no cognitive impairment. There must also be no formal diagnosis or clinical input of dementia (e.g. when the clinical team suspect that the patient has dementia and he/she is referred to a memory assessment service).

Patients will be grouped as ‘undetermined’ if *all* information is available showing that they present some degree of delirium symptoms without meeting full DSM-5 diagnostic criteria for delirium. Thus, this is likely subsyndromal or resolving delirium. Patients who are currently undergoing clinical investigation for dementia but where no outcome has been decided will also be classified as undetermined.

### Data management and storage

Data from the reference standard and index assessment, as well as medical information and IQCODE will be recorded on paper Case Report Forms. The data on the Case Report Forms will be transcribed by the researchers into a secure database created by the Edinburgh Clinical Trials Unit.

### Sample size calculation

Of the 500 patients recruited, 20% would be expected to have delirium [3]. Sample sizes were obtained using a normal approximation to the binomial distribution to estimate a 95% confidence interval (CI) for measures of diagnostic accuracy for delirium. The anticipated confidence interval widths are shown in Table 2 for a range of levels of diagnostic test performance.

**Table 2:** Precision of sensitivity and specificity estimation (delirium versus control comparison)

Sample size	Parameter	True level of parameter	95% CI width
500	Sensitivity	0.5	±0.098
	Sensitivity	0.7	±0090
	Sensitivity	0.9	±0.059
	Specificity	0.5	±0.049
	Specificity	0.7	±0.045
	Specificity	0.9	±0.029

The sensitivity precision in the delirium versus dementia comparison will be identical to the details outlined above, as the size of the delirium target group is unchanged. The dementia group is similar in size to the delirium group, hence the precision of specificity estimation in this comparison will be similar to that given for *sensitivity*. These estimates are based on a delirium prevalence of 20%

In a sample of 500 patients, as per the original study protocol, specificity for the delirium versus no delirium comparison and the delirium versus dementia comparison can be estimated precisely in all scenarios; sensitivity can be measured precisely when the diagnostic performance is good, and moderately precisely in other scenarios (Table 2).

### Statistical analysis plan

The statistical analysis plan was prepared by the study statisticians (CJW, VA).

#### Primary objective

The diagnostic accuracy of the DelApp versus the reference standard will be determined using sensitivity, specificity, and positive and negative predictive values. The DelApp total score will be analysed in a univariate logistic regression and ROC curve analysis. From this we will obtain a measure of the discriminating ability of the DelApp score, by calculating the Area Under the ROC Curve (AUC). We will also calculate the optimal cut-off using Youden’s index [23] to assess if the a priori DelApp cut-off scores are appropriate. These a priori DelApp cut points are 8 for discriminating delirium from the whole sample, and 6 for discriminating between delirium and dementia [2].

#### Secondary objectives

Using Spearman correlations, we will evaluate the association between DelApp scores with delirium severity as measured with the DRS-R98 (secondary objective A) and tests of attention (digit span, months of the year backward, days of the week backward, and Vigilance A’; secondary objective B) to investigate construct validity.

To assess criterion validity, the association between DelApp total scores and length of stay, mortality at 12 weeks after study participation and discharge location will be evaluated with normal linear models (secondary objective C).

To explore the performance of the DelApp in tracking change in attentional functioning and delirium status, changes in test scores over time (from patients who have undergone repeat assessments), rescaled to a common range of values to facilitate comparison between tests, will be presented in descriptive statistics and graphically, comparing baseline with each of the other time points (secondary objective D).

Inter-rater reliability will be evaluated using the Kappa statistic and its 95% CI (secondary objective E).

Participants with missing data on the primary outcome measures will be removed from the statistical analysis. In case of a high proportion of missing data we will consider an imputation approach.

All statistical tests will be two-sided and will be performed using a 0.05 significance level and 95% (two-sided) CIs will be presented. There will be no statistical adjustment for multiplicity of analyses; there is a single primary analysis and the interpretation of secondary analyses will be suitably cautious to reflect the large number of variables considered.

For all statistical analyses, the primary analyses will be unadjusted. We will also present secondary analyses adjusted for age, sex, centre and IQCODE.

### Monitoring and safety

This study is being monitored by the Academic and Clinical Central Office for Research and Development (ACCORD). The assessors will record any adverse events, serious adverse events, adverse device effects and serious adverse device effects that might occur during the assessments in line with the ACCORD guidelines. When serious, these events will also be reported to the Medicines and Healthcare products Regulatory Agency.

### Training

Researchers at the University of Edinburgh and University of Glasgow were extensively trained by the Principal Investigator, Co-Investigators and a post-doctoral Research Fellow (ZT) working on the project. All researchers were certified in Good Clinical Practice and received comprehensive training, using training videos on delirium, observation of clinicians on hospital wards, role-playing scenarios and introductory visits to each ward. Furthermore, appropriate training on the use of electronic software which maintains medical records was given where appropriate. The researchers will be observed by experienced clinicians and/or the Research Fellow at regular intervals to ensure quality of assessments.

### Dissemination

After data analysis the results of this study will be published in appropriate medical academic journals. Moreover, we aim to disseminate this study and its results at academic delirium related conferences including conferences organised by the European Delirium Association and American Delirium Society. Patients who explicitly express a wish to be informed about the research outcome will be contacted and offered to receive an article or poster with a lay summary of the study. All academic papers and posters will be written by the core members of the research team.

## Discussion

There is a need for objective assessment tools for delirium which are practical and feasible for use in elderly acute care settings. We developed a novel neuropsychological test for assessing arousal and sustained attention in delirium, implemented on a smartphone platform (DelApp). Computer-based tests have potential advantages over paper and pencil alternatives in clinical practice and at the bedside. These include standardised instructions, controlled presentation of stimuli and objective scoring methods. Smartphone-based applications are increasingly being used in healthcare and computer-based cognitive testing is now feasible in routine clinical practice [33].

The DelApp provides an easy-to-administer smartphone test which has potential to assist assessment of delirium at the bedside. It is important to evaluate new methods for delirium assessment such as DelApp in studies that: (i) employ rigorous diagnostic test accuracy methods with explicit operationalised diagnostic criteria (i.e. reference standard); (ii) describe a clear and transparent rationale for group allocation, including those in whom there is diagnostic uncertainty; and (iii) evaluate tools in real-world settings, including in patients with potential difficulties in communication and overlapping symptom profiles such as dementia. The present study protocol was designed to meet these criteria.

This study will give new data on performance of DelApp in detection of delirium in a representative cohort of older hospital patients. Further, the research value of DelApp for detecting inattention but also for grading severity and tracking change in attentional function and delirium over time will be evaluated.

## Additional files

Additional file 1: This document presents instructions and scoring procedures for the DelApp arousal and attention assessments. **Table S1.** DelApp assessment and scoring procedure (i.e. arousal and attention assessment). **Figure S1.** Schematic display of DelApp arousal assessment and scoring. (PDF 124 kb)

Additional file 2: This document presents a table (**Table S2**) that provides additional detail on the instructions and scoring methods of the neuropsychological tests used in the reference standard assessment. (PDF 129 kb)

## Abbreviations

ACCORD: Academic and clinical central office for research and development
AMT10: Abbreviated Mental Test-10
DRS-R98: Delirium Rating Scale-Revised 98
DSM-5: Diagnostic and Statistical Manual of Mental Disorders-5
EDTB: Edinburgh Delirium Test Box
IQCODE: Informant Questionnaire on Cognitive Decline in the Elderly
OMCT: Orientation-Memory-Concentration Test
OSLA: Observational Scale of Level of Arousal
RASS: Richmond Agitation-Sedation Scale
ROC: Receiver Operating Characteristic
