# Supplementary material for: Diagnostic test accuracy of a novel smartphone application for the assessment of attention deficits in delirium in older hospitalised patients: a prospective cohort study protocol
Source: BMC Geriatr. 2018 Sep 17;18:217. doi: 10.1186/s12877-018-0901-5 (PMC6142423; doi:10.1186/s12877-018-0901-5)

Table S1. DelApp assessment and scoring procedure

| Assessment | Trial | Instructions for assessor | Scoring |
|---|---|---|---|
| 1. Arousal | Question 1 | Is the participant responsive or arousable, or does the participant open eyes to speech (say their name) or touch on shoulder for more than 10 seconds? | Yes (1 point)<br>No (0 points) |
| | Question 2 (only if question 1 is "no") | Does the participant open eyes briefly (less than 10 seconds) to speech or touch on the shoulder? | Yes (1 point)<br>No (0 points)<br>N.B. if Q1 yes, this item will be scored 1 point. |
| | Question 3 | Can the participant say their name, or (if not) obey a one stage command, for example lifting one arm? | Yes (1 point)<br>No (0 points) |
| | Question 4 | Can the participant follow an object with their eyes for 5 seconds? | Yes (1 point)<br>No (0 points) |
| 2. Visual pre-test | Identifying shape | Say to the participant:<br><br>*"In a moment a white star will be shown on the screen of the phone. Can you tell me when you see this star appear?"*<br><br>Did the participant observe the star? | Not scored. |

| | | | |
|---|---|---|---|
| 3. Sustained attention task | Trial 1 (practice), 2 and 3 | Say to the participant:<br><br>"*You will see a series of stars appear on the screen. I want you to keep looking at the screen and count the stars. You should keep counting until I ask you how many stars you counted. Are you ready?*"<br><br>Press OK when you are ready to continue with the practice. | Practice trial is not scored. For trials 2 and 3, 1 point is given for each correct answer. |
| | Trials 4 to 7 | Say to the participant:<br><br>"*As before, every now and then a white star will appear on the screen. I want you to keep looking at the screen and count how many stars you see. You will also see some small triangles appearing on the screen. Please try ignoring these triangles and just keep counting the stars as before. You should keep counting until I ask you how many stars you counted.*<br>*Are you ready?*" | 1 point for each correct answer. |
| | | | DelApp total score: 0-10 (10 = good performance) |

Figure S1. Schematic display of DelApp arousal assessment and scoring.

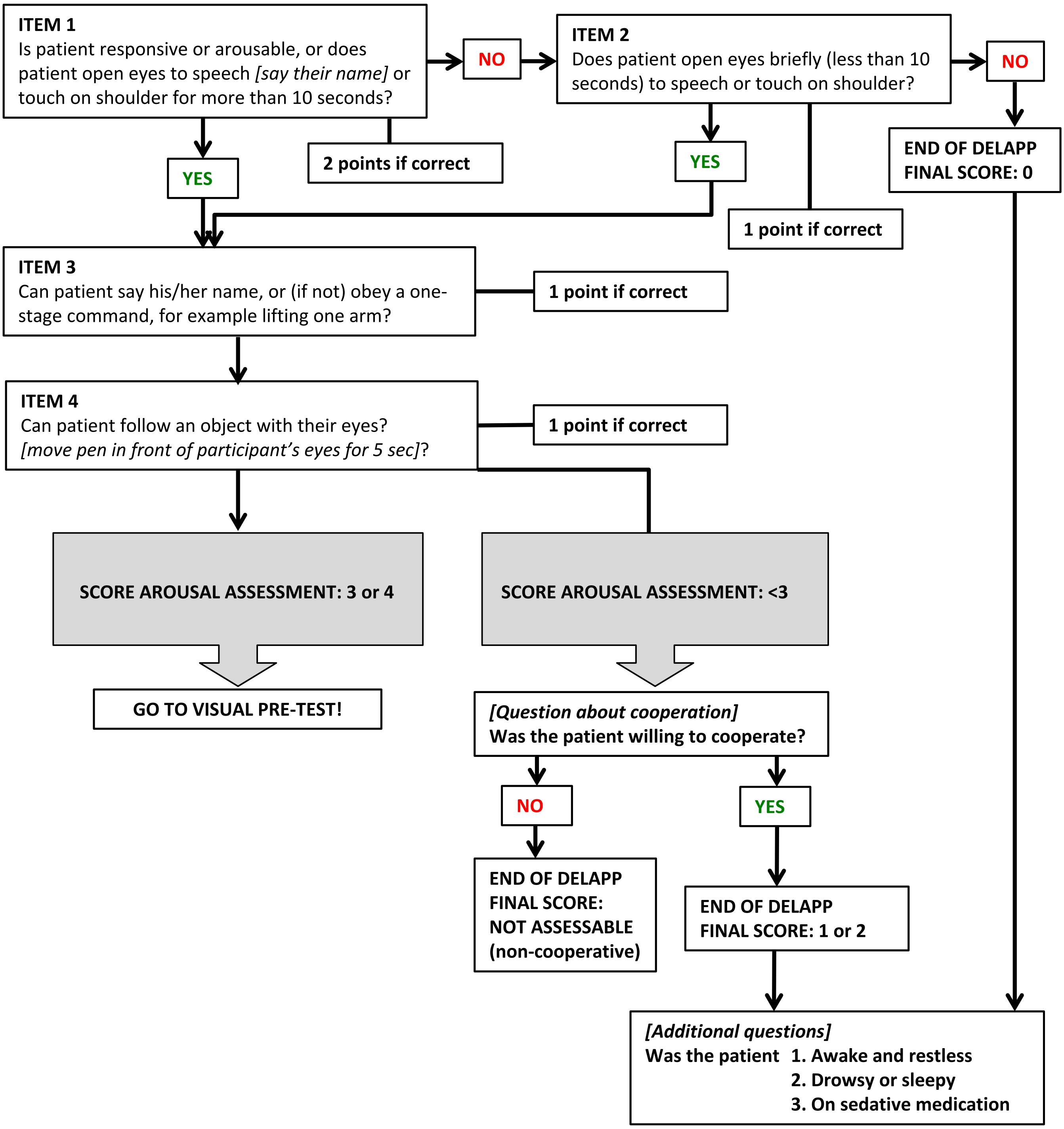

Supplement: Supplementary file 1 — This document presents instructions and scoring procedures for the DelApp arousal and attention assessments. Table S1. DelApp assessment and scoring procedure (i.e. arousal and attention assessment). Figure S1. Schematic display of DelApp arousal assessment and scoring. (PDF 124 kb) [file 12877_2018_901_MOESM1_ESM.pdf]
