# Supplementary material for: Diagnostic test accuracy of a novel smartphone application for the assessment of attention deficits in delirium in older hospitalised patients: a prospective cohort study protocol
Source: BMC Geriatr. 2018 Sep 17;18:217. doi: 10.1186/s12877-018-0901-5 (PMC6142423; doi:10.1186/s12877-018-0901-5)
Supplement: Supplementary file 2 — This document presents a table (Table S2) that provides additional detail on the instructions and scoring methods of the neuropsychological tests used in the reference standard assessment. (PDF 129 kb) [file 12877_2018_901_MOESM2_ESM.pdf]

Table S2. Reference standard assessment: Cognitive tests instructions and scoring.

AMT10: Abbreviated Mental Test 10; DRS-R98: Delirium Rating Scale Revised 98; DSM-5: Diagnostic Statistical Manual-5; Short OMCT: Short Orientation Memory and Concentration Task.

**Test**	**Instructions**	**Scoring procedure and additional comments**
Short OMCT	Q1. Year<br>"*What year is it now?*"	Scoring: 4 points if correct, 0 points if incorrect.<br>The exact year must be given. An incomplete but correct numerical response is acceptable (i.e. 15 for 2015). Do not correct or reorient the participant in case of an incorrect response, be as neutral as possible, unless they directly ask you (such as "*I am not sure, is it 2000?*").
	Q2. Month<br>*"What month is it now?"*	Scoring: 3 points if correct, 0 points if incorrect.<br>The exact month must be given. An acceptable response is either the name of the month, or its numerical equivalent (i.e. 12 for December). Do not correct the participant in case of an incorrect answer, be as neutral as possible, unless they directly ask you (such as "*I am not sure, can you tell me the month?*").
	Q3. Instant address recall<br>*"I'm going to read you a name and address that I'd like you to repeat after me. We'll be doing that a couple of times, so you have a chance to*	The researcher reads out the address three times and the participant is asked to repeat the address after hearing each repetition. If the participant starts repeating along with you, ask them to wait until you have finished.

	learn it. Is that clear? Ok: Mr John Brown, 42 West Street, Gateshead".	If no correct response is given after 3 trials, record “NO”. Say:<br>“*Ok, try to remember it, as I am going to ask you later about it.*”<br><br>This item is not scored.
	Q4. Time<br>“*Without looking at your watch, can you tell me approximately what time it is now?*”	Scoring: 3 points if correct, 0 points if incorrect.<br>Allow +/- 1 hour. If the participant's response is unclear (such as “*morning*”), they should be prompted to give a more specific time.
	Q5. Counting 20 down to 1<br>*“Can you count backwards from 20 down to 1?*”	Scoring: 4 points if correct, 2 points if 1 error, 0 points if 2 errors.<br>All numbers must be given in the correct order. For each incorrectly placed number, score one error. Below are scoring instructions for possible scenarios:<br>• If the participant starts counting forward in the middle of the task (such as “20 - *19 - 18 - 17 - 18 - 19 –20*”), score the maximum number of errors (0 points) and do not prompt the participant to try again, but terminate the task.<br>• If the participant skips one number, but finishes the task otherwise correctly, score one error for the skipped number.<br>• If the participant skips two or more numbers, score the maximum number of errors (0

		points).<br>• If the participant self-corrects (such as "*20 – 19 – 18 – 17 – 16 – 15 – 13 … uh no 14 – 13 - 12 etc."*) score one error.<br>• If a participant is very drowsy and falls asleep during the task, score 2 errors, terminate the task and gently wake them up to continue with the next task.
	Q6. Months of the year backwards<br>"*Could you please tell me the months of the year in reverse order, starting with December?*"	Scoring: 4 points if correct, 2 points if 1 error, 0 points if 2 errors.<br>All months must be given in the correct order. For each incorrectly placed month, score one error. Below are scoring instructions for possible scenarios:<br>• If the participant starts counting forward in the middle of the task (such as "*December - November - October - September - October - November –December*"), score the maximum number of errors (0 points) and do not prompt the participant to try again, but terminate the task.<br>• If the participant skips one month, but finishes the task otherwise correctly, score one error for the skipped month.<br>• If the participant skips two or more months, score the maximum number of errors (0 points).<br>• If the participant self-corrects (such as "*December – November – October –*

		November… uh no September – August etc.") score one error.<br>• If a participant is very drowsy and falls asleep during the task, score the maximum number of errors (0 points), terminate the task and gently wake them up to continue with the next task.<br>• If the participant waits in between correctly produced months, a wait time of up to 90 seconds is allowed and should be scored as correct.
	Q7. Delayed address recall<br>"*Earlier I have given you a name and an address. Can you try to remember the name and address and tell me what it was?*"	The participant should repeat the complete address that was given in Q3. All underlined elements should be given. Maximum possible score = 10; subtract 2 points for each missing element.
AMT10	Q1. Age<br>"*May I ask how old you are*?"	Score 1 if correct, score 0 if incorrect.<br>Note the answer and check later with medical records. Exact age (in years) must be given.
	Q2. – Q4. Part of OMCT, do not repeat	See OMCT Q1 – Q4.<br>Score 1 if correct, score 0 if incorrect.
	Q5. Place<br>"*Do you know where we are right now*?"	Score 1 if correct, score 0 if incorrect.<br>• The name of the hospital must be given. If participant replies vaguely (e.g. "*Hospital*"),

		one prompt to elicit a more detailed answer is allowed.<br>• If the participant says part of the name of the hospital, but indicating that they know where they are (e.g. "Royal" or "*Infirmary Edinburgh*"), score as correct. However, if they say "*Royal Infirmary London*" or make another error, score as incorrect.
	Q6. Month. Part of OMCT, do not repeat	Score 1 if correct, score 0 if incorrect.<br>See OMCT Q2.
	Q7. Date of birth<br>"*What is your date of birth*?"	Score 1 if correct, score 0 if incorrect.<br>Note the answer and check later with medical records. The exact date of birth must be given.
	Q8. World War II<br>"*When was the Second World War?*"	Score 1 if correct, score 0 if incorrect.<br>Start and end date must be given and both correct. One prompt is allowed, such as "*and when was the end*?" if the participant only responds e.g. "*1939*".
	Q9. Prime Minister<br>"*Can you tell me the name of the current prime minister*?"	Score 1 if correct, score 0 if incorrect.<br>This must be the name of current Prime Minister (i.e. Theresa May). Either first and last name, or only last name is scored as correct.
Digit Span Forward	*"I'm going to read out a sequence of numbers to you. Listen carefully, and when I am finished, I*	Maximum possible score = 3 (sequence length of 3, 4 and 5 digits)<br>• Start with a practice example. If you are confident that the participant understands the

	want you to say them right after me. Just say what I say. For example, if I said 1-7, you would also say 1-7. OK?"	instructions, proceed with the test items.<br>• Read digit sequences from part "digit span forward" from record form at a rate of one digit per second.<br>• Provide no assistance with items and do not repeat items unless it is very clear that the participant has problems with hearing.<br>• Record responses verbatim.<br>• Score correct (1 point) if one OR both sequences of a given length (e.g., 3 digits) is correctly repeated.<br>• Go through items 1-4 (sequences of 3 and 4 digits) regardless of response. After this, discontinue after a score of 0 on *both* trials of any sequence length.
Digit Span Backwards	*"Now I am going to say some more numbers. But this time when I stop, I want you to say them backwards. For example, if I say 2-4, you would say 4-2. OK?"*	Maximum possible score = 2 (sequence length of 3 and 4 digits)<br>• Start with a practice example. If you are confident that the participant understands the instructions, proceed with the test items.<br>• Read digit sequences from "digit span backwards" at a rate of one digit per second.<br>• If participant responds correctly *say "that's right" and* proceed to trial 1.<br>• If participant responds incorrectly, reminds them of the task instructions. Do not provide any assistance on this second example. Proceed to next trial regardless of whether the

		participant scores correctly.<br>• Read digits at a rate of 1 per second.<br>• Provide no assistance with items in test and do not repeat unless if the participant has problems hearing.<br>• Record responses verbatim.<br>• Score correct (1 point) if one OR both sequences of a given length (e.g., 3 digits) is correctly repeated.<br>• Discontinue if participant gives incorrect responses for both 3-digit sequences.
Days of week backwards	"*Can you tell me the days of the week backwards, starting with Sunday*?"	Don't give any prompts throughout the task, no matter what type of error the participant makes. The participant only gets a point for this task when all days are given in the correct backwards order, hence, no error is allowed. For each incorrectly placed day, score this item as incorrect. Below are scoring instructions for possible scenarios:<br>• If the participant starts counting forward in the middle of the task (such as "*Sun - Sat - Fri - Thu - Wed - Thu - Fri – Sat*"), score as incorrect and do not prompt the participant to try again, but terminate the task.<br>• If the participant skips one day, but finishes the task otherwise correctly, score as incorrect.

		• If the participant skips two or more days, score as incorrect.<br>• If the participant self-corrects themselves (such as "*Sun - Sat – Fri – Thu – Wed – Tue – Sun – ... uh no Mon – Sun.*") score as incorrect.<br>• If a participant is very drowsy and falls asleep during the task, score as incorrect, terminate the task and gently wake them up to continue with the next task.
DRS-R98	Visuospatial ability: Identify two objects within the participant's bed space and ask which object is closer. Also ask<br>*"Does the room look tilted?"*<br>And consider the placement of initials and signature on the consent form.	If the participant cannot identify which item is closest to them, states that the room is tilted or has placed their signature and initials in the incorrect place on the consent form, score as "visuospatial disturbance present".
	Hallucinations / Delusions<br>If the participant is able to speak, say:<br>*"Sometimes people in hospital develop strange experiences, such as seeing things that other people can't see. Has this happened to you? Also, sometimes people can become afraid of the*	Score hallucinations/delusions present or absent depending on the participant's response. If participant indicates that they have had hallucinations or delusions, ask if they can remember if this was in the past 24 hours or longer ago.

	people or things around them. Have you experienced this? Do you have beliefs that other people tell you aren't true?"	
